# 6-Hydr­oxy-3-(hydroxy­imino)indolin-2-one

**DOI:** 10.1107/S1600536809034321

**Published:** 2009-09-05

**Authors:** Hui-ling Yu

**Affiliations:** a Yibin Vocational & Technical College, Si chuan, People’s Republic of China

## Abstract

In the title compound, C_8_H_6_N_2_O_3_, the indol-2-one system is almost planar [maximum deviation = 0.010 (3) Å]. In the crystal structure, inter­molecular N—H⋯O, O—H⋯N and O—H⋯O hydrogen bonds link the mol­ecules into a three-dimensional network. π–π contacts between the indole ring systems [centroid–centroid distances = 3.494 (1), 3.731 (1) and 3.736 (1) Å] may further stabilize the structure.

## Related literature

For the biological and pharmacological properties of isatin-3-oxime derivatives, see: Pinto *et al.* (2008 ▶). For bond-length data, see: Allen *et al.* (1987 ▶).
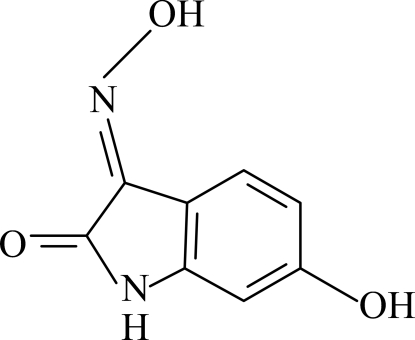


## Experimental

### 

#### Crystal data

C_8_H_6_N_2_O_3_
*M* *_r_* = 178.15
Monoclinic, 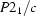

*a* = 7.4160 (15) Å
*b* = 7.1240 (14) Å
*c* = 14.111 (3) Å
β = 95.21 (3)°
*V* = 742.4 (3) Å^3^
*Z* = 4
Mo *K*α radiation
μ = 0.13 mm^−1^
*T* = 294 K
0.30 × 0.30 × 0.10 mm

#### Data collection

Enraf–Nonius CAD-4 diffractometer
Absorption correction: ψ scan (North *et al.*, 1968 ▶) *T*_min_ = 0.963, *T*_max_ = 0.988
2787 measured reflections
1350 independent reflections
994 reflections with *I* > 2σ(*I*)
*R*_int_ = 0.068
3 standard reflections frequency: 120 min intensity decay: 1%

#### Refinement

*R*[*F* ^2^ > 2σ(*F* ^2^)] = 0.061
*wR*(*F* ^2^) = 0.172
*S* = 1.00
1350 reflections
118 parameters
H-atom parameters constrained
Δρ_max_ = 0.44 e Å^−3^
Δρ_min_ = −0.50 e Å^−3^

### 

Data collection: *CAD-4 Software* (Enraf–Nonius, 1989 ▶); cell refinement: *CAD-4 Software*; data reduction: *XCAD4* (Harms & Wocadlo, 1995 ▶); program(s) used to solve structure: *SHELXS97* (Sheldrick, 2008 ▶); program(s) used to refine structure: *SHELXL97* (Sheldrick, 2008 ▶); molecular graphics: *ORTEP-3 for Windows* (Farrugia, 1997 ▶); software used to prepare material for publication: *SHELXL97* and *PLATON* (Spek, 2009 ▶).

## Supplementary Material

Crystal structure: contains datablocks global, I. DOI: 10.1107/S1600536809034321/hk2760sup1.cif

Structure factors: contains datablocks I. DOI: 10.1107/S1600536809034321/hk2760Isup2.hkl

Additional supplementary materials:  crystallographic information; 3D view; checkCIF report

## Figures and Tables

**Table 1:** Hydrogen-bond geometry (Å, °)

*D*—H⋯*A*	*D*—H	H⋯*A*	*D*⋯*A*	*D*—H⋯*A*
N1—H1*A*⋯O2^i^	0.86	2.05	2.854 (4)	156
O1—H1*C*⋯N1^ii^	0.96	2.52	3.466 (4)	168
O3—H3*A*⋯O2^iii^	0.82	2.00	2.753 (3)	152

Symmetry codes: (i) 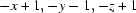; (ii) 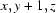; (iii) 
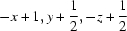
.

**Fig. 1.**
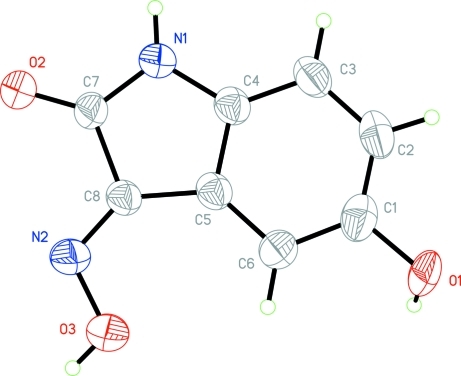
The molecular structure of the title molecule, with the atom-numbering scheme. Displacement ellipsoids are drawn at the 50% probability level.

## supplementary crystallographic information

### Comment

The title compound is one kind of important isatin-3-oxime derivatives, which displays diverse biological and pharmacological properties (Pinto *et al.*,  2008). We report herein its crystal structure.

In the molecule of the title compound, (Fig. 1), the bond lengths (Allen *et al.*,  1987) and angles are within normal ranges. The indole ring system is planar with a maximum deviation of -0.010 (3) Å for atom C2. Atoms O1, O2, O3 and N2 are 0.005 (3), -0.059 (3), -0.184 (3) and -0.085 (3) Å away from the plane of the indole ring system, respectively.

In the crystal structure, intermolecular N-H···O, O-H···N and O-H···O hydrogen bonds (Table 1) link the molecules into a three-dimensional network, in which they may be effective in the stabilization of the structure. The π–π contacts between the indole rings, Cg1—Cg1^i^, Cg2—Cg2^ii^ and Cg1—Cg2^i^ [symmetry codes: (i) 1 - x, 1 - y, -z, (ii) 2 - x, 1 - y, -z, where Cg1 and Cg2 are centroids of the rings (N1/C4/C5/C7/C8) and (C1-C6), respectively] may further stabilize the structure, with centroid-centroid distances of 3.494 (1), 3.731 (1) and 3.736 (1) Å, respectively.

### Experimental

For the preparation of the title compound, 2-(hydroxyimino)-N-(3-hydroxyphenyl)- acetamide (1 mmol), 1-n-butyl-3-methylimidazolium chloride (0.5 mmol) and 2,2,2-trifluoroacetic acid (0.05 mmol) were added into a sealed flask. The mixture was stirred for 90 min and the temperature maintained at 408 K. After the completion of reaction, ether was used to extract organic compounds from the ionic liquid phase, and the combined organic layers were concentrated under reduced pressure. Product purification was performed by column chromatography. Crystals suitable for X-ray analysis were obtained by dissolving the title compound (0.1 g) in ethyl acetate (10 ml) and evaporating the solvent slowly at room temperature for 2 d.

### Refinement

H atoms were positioned geometrically with N-H = 0.86 Å (for NH), O-H = 0.82 and 0.96 Å (for OH) and C-H = 0.93 Å for aromatic H atoms, respectively, and constrained to ride on their parent atoms, with U_iso_(H) = xU_eq_(C,N,O), where x = 1.5 for OH H and x = 1.2 for all other H atoms.

### Crystal data

**Table:**

C_8_H_6_N_2_O_3_	*F*(000) = 368
*M**_r_* = 178.15	*D*_x_ = 1.594 Mg m^−^^3^
Monoclinic, *P*2_1_/*c*	Mo *K*α radiation, λ = 0.71073 Å
Hall symbol: -P 2ybc	Cell parameters from 25 reflections
*a* = 7.4160 (15) Å	θ = 9–13°
*b* = 7.1240 (14) Å	µ = 0.13 mm^−^^1^
*c* = 14.111 (3) Å	*T* = 294 K
β = 95.21 (3)°	Block, yellow
*V* = 742.4 (3) Å^3^	0.30 × 0.30 × 0.10 mm
*Z* = 4	

### Data collection

**Table:**

Enraf–Nonius CAD-4 diffractometer	994 reflections with *I* > 2σ(*I*)
Radiation source: fine-focus sealed tube	*R*_int_ = 0.068
graphite	θ_max_ = 25.3°, θ_min_ = 2.8°
ω/2θ scans	*h* = 0→8
Absorption correction: ψ scan (North *et al.*, 1968)	*k* = −8→8
*T*_min_ = 0.963, *T*_max_ = 0.988	*l* = −16→16
2787 measured reflections	3 standard reflections every 120 min
1350 independent reflections	intensity decay: 1%

### Refinement

**Table:**

Refinement on *F*^2^	Primary atom site location: structure-invariant direct methods
Least-squares matrix: full	Secondary atom site location: difference Fourier map
*R*[*F*^2^ > 2σ(*F*^2^)] = 0.061	Hydrogen site location: inferred from neighbouring sites
*wR*(*F*^2^) = 0.172	H-atom parameters constrained
*S* = 1.00	*w* = 1/[σ^2^(*F*_o_^2^) + (0.08*P*)^2^ + 0.6*P*] where *P* = (*F*_o_^2^ + 2*F*_c_^2^)/3
1350 reflections	(Δ/σ)_max_ < 0.001
118 parameters	Δρ_max_ = 0.44 e Å^−^^3^
0 restraints	Δρ_min_ = −0.50 e Å^−^^3^

### Special details

**Table:**

Geometry. All esds (except the esd in the dihedral angle between two l.s. planes) are estimated using the full covariance matrix. The cell esds are taken into account individually in the estimation of esds in distances, angles and torsion angles; correlations between esds in cell parameters are only used when they are defined by crystal symmetry. An approximate (isotropic) treatment of cell esds is used for estimating esds involving l.s. planes.
Refinement. Refinement of F^2^ against ALL reflections. The weighted R-factor wR and goodness of fit S are based on F^2^, conventional R-factors R are based on F, with F set to zero for negative F^2^. The threshold expression of F^2^ > 2sigma(F^2^) is used only for calculating R-factors(gt) etc. and is not relevant to the choice of reflections for refinement. R-factors based on F^2^ are statistically about twice as large as those based on F, and R- factors based on ALL data will be even larger.

### Fractional atomic coordinates and isotropic or equivalent isotropic displacement parameters (Å^2^)

**Table:**

	*x*	*y*	*z*	*U*_iso_*/*U*_eq_	
O1	0.9722 (3)	0.3850 (3)	0.61373 (16)	0.0540 (7)	
H1C	0.8846	0.4824	0.5992	0.081*	
O2	0.5104 (3)	−0.3981 (3)	0.38450 (15)	0.0518 (6)	
O3	0.6534 (3)	0.1382 (3)	0.29758 (15)	0.0573 (7)	
H3A	0.6170	0.1652	0.2427	0.086*	
N1	0.6497 (4)	−0.2911 (4)	0.52693 (17)	0.0446 (7)	
H1A	0.6331	−0.3880	0.5615	0.054*	
N2	0.6071 (3)	−0.0423 (4)	0.31580 (17)	0.0443 (7)	
C1	0.8929 (4)	0.2147 (5)	0.5948 (2)	0.0489 (8)	
C2	0.8858 (4)	0.0912 (5)	0.6684 (2)	0.0504 (9)	
H2B	0.9338	0.1245	0.7292	0.060*	
C3	0.8071 (4)	−0.0837 (5)	0.6524 (2)	0.0458 (8)	
H3B	0.8021	−0.1699	0.7015	0.055*	
C4	0.7367 (4)	−0.1255 (4)	0.5612 (2)	0.0381 (7)	
C5	0.7418 (4)	0.0035 (4)	0.48642 (18)	0.0369 (7)	
C6	0.8213 (4)	0.1782 (4)	0.5024 (2)	0.0424 (7)	
H6A	0.8263	0.2660	0.4539	0.051*	
C7	0.5958 (4)	−0.2790 (4)	0.4338 (2)	0.0387 (7)	
C8	0.6537 (4)	−0.0880 (4)	0.40277 (19)	0.0352 (7)	

### Atomic displacement parameters (Å^2^)

**Table:**

	*U*^11^	*U*^22^	*U*^33^	*U*^12^	*U*^13^	*U*^23^
O1	0.0682 (15)	0.0357 (12)	0.0553 (14)	−0.0148 (11)	−0.0103 (11)	−0.0125 (10)
O2	0.0762 (15)	0.0374 (13)	0.0403 (12)	−0.0061 (11)	−0.0038 (11)	−0.0018 (10)
O3	0.0793 (16)	0.0455 (14)	0.0451 (13)	−0.0082 (12)	−0.0054 (11)	0.0099 (10)
N1	0.0608 (16)	0.0344 (14)	0.0371 (13)	0.0006 (12)	−0.0042 (11)	0.0031 (11)
N2	0.0558 (16)	0.0358 (14)	0.0411 (14)	0.0023 (12)	0.0034 (12)	0.0048 (11)
C1	0.0478 (18)	0.0425 (18)	0.0549 (19)	0.0001 (15)	−0.0036 (15)	−0.0128 (15)
C2	0.0521 (18)	0.058 (2)	0.0383 (16)	0.0025 (16)	−0.0081 (13)	−0.0096 (16)
C3	0.0490 (18)	0.051 (2)	0.0354 (16)	0.0054 (15)	−0.0063 (13)	0.0016 (14)
C4	0.0381 (15)	0.0380 (16)	0.0370 (15)	0.0066 (13)	−0.0027 (12)	−0.0006 (12)
C5	0.0402 (15)	0.0362 (16)	0.0335 (15)	0.0065 (13)	0.0001 (11)	−0.0005 (12)
C6	0.0487 (17)	0.0354 (16)	0.0428 (16)	0.0011 (14)	0.0022 (13)	−0.0016 (13)
C7	0.0478 (16)	0.0317 (15)	0.0353 (15)	0.0039 (13)	−0.0028 (12)	−0.0034 (12)
C8	0.0395 (15)	0.0333 (15)	0.0321 (14)	0.0050 (12)	0.0000 (11)	0.0010 (12)

### Geometric parameters (Å, °)

**Table:**

O1—C1	1.364 (4)	C1—C6	1.387 (4)
O1—H1C	0.9600	C2—C3	1.386 (5)
O2—C7	1.234 (3)	C2—H2B	0.9300
O3—H3A	0.8200	C3—C4	1.376 (4)
N1—C4	1.409 (4)	C3—H3B	0.9300
N1—C7	1.342 (4)	C4—C5	1.402 (4)
N1—H1A	0.8600	C5—C6	1.387 (4)
N2—O3	1.361 (3)	C5—C8	1.451 (4)
N2—C8	1.286 (4)	C6—H6A	0.9300
C1—C2	1.365 (5)	C7—C8	1.504 (4)
			
C1—O1—H1C	109.2	C3—C4—C5	121.9 (3)
N2—O3—H3A	109.5	C3—C4—N1	128.7 (3)
C4—N1—H1A	124.2	C5—C4—N1	109.4 (2)
C7—N1—C4	111.6 (2)	C6—C5—C4	120.4 (3)
C7—N1—H1A	124.2	C6—C5—C8	133.5 (3)
C8—N2—O3	111.6 (3)	C4—C5—C8	106.1 (3)
O1—C1—C2	118.0 (3)	C5—C6—C1	116.2 (3)
O1—C1—C6	118.2 (3)	C5—C6—H6A	121.9
C2—C1—C6	123.8 (3)	C1—C6—H6A	121.9
C1—C2—C3	120.0 (3)	O2—C7—N1	126.8 (3)
C1—C2—H2B	120.0	O2—C7—C8	127.1 (3)
C3—C2—H2B	120.0	N1—C7—C8	106.0 (2)
C4—C3—C2	117.7 (3)	N2—C8—C5	136.4 (3)
C4—C3—H3B	121.1	N2—C8—C7	116.6 (3)
C2—C3—H3B	121.1	C5—C8—C7	106.8 (2)
			
O1—C1—C2—C3	−179.7 (3)	C2—C1—C6—C5	−1.1 (5)
C6—C1—C2—C3	1.4 (5)	C4—N1—C7—O2	−177.1 (3)
C1—C2—C3—C4	−0.5 (5)	C4—N1—C7—C8	0.5 (3)
C2—C3—C4—C5	−0.6 (4)	O3—N2—C8—C5	0.5 (5)
C2—C3—C4—N1	−179.6 (3)	O3—N2—C8—C7	175.0 (2)
C7—N1—C4—C3	178.8 (3)	C6—C5—C8—N2	−4.9 (6)
C7—N1—C4—C5	−0.3 (3)	C4—C5—C8—N2	175.2 (3)
C3—C4—C5—C6	0.9 (4)	C6—C5—C8—C7	−179.7 (3)
N1—C4—C5—C6	−180.0 (3)	C4—C5—C8—C7	0.3 (3)
C3—C4—C5—C8	−179.2 (3)	O2—C7—C8—N2	1.1 (5)
N1—C4—C5—C8	0.0 (3)	N1—C7—C8—N2	−176.6 (3)
C4—C5—C6—C1	0.0 (4)	O2—C7—C8—C5	177.2 (3)
C8—C5—C6—C1	−180.0 (3)	N1—C7—C8—C5	−0.5 (3)
O1—C1—C6—C5	−180.0 (3)		

### Hydrogen-bond geometry (Å, °)

**Table:**

*D*—H···*A*	*D*—H	H···*A*	*D*···*A*	*D*—H···*A*
N1—H1A···O2^i^	0.86	2.05	2.854 (4)	156
O1—H1C···N1^ii^	0.96	2.52	3.466 (4)	168
O3—H3A···O2^iii^	0.82	2.00	2.753 (3)	152

Symmetry codes: (i) −*x*+1, −*y*−1, −*z*+1; (ii) *x*, *y*+1, *z*; (iii) −*x*+1, *y*+1/2, −*z*+1/2.
